# Inferring fine-scale spatial structure of the brown bear (*Ursus arctos*) population in the Carpathians prior to infrastructure development

**DOI:** 10.1038/s41598-019-45999-y

**Published:** 2019-07-01

**Authors:** Ancuta Fedorca, Isa-Rita M. Russo, Ovidiu Ionescu, Georgeta Ionescu, Marius Popa, Mihai Fedorca, Alexandru Lucian Curtu, Neculae Sofletea, Gary M. Tabor, Michael W. Bruford

**Affiliations:** 1 National Institute for Research and Development in Forestry Marin Dracea, Brasov, 500040 Closca Street 13, Romania; 2 Faculty of Silviculture and Forest Engineering, Transilvania University of Brasov, Brasov, 500123 Beethoven Lane 1, Romania; 3 Cardiff School of Biosciences, Sir Martin Evans Building, Cardiff University, Museum Avenue, Cardiff, CF10 3AX United Kingdom; 4 Center for Large Landscape Conservation, 303 W Mendenhall St #4, Bozeman, MT 59715 USA

**Keywords:** Evolutionary biology, Molecular ecology, Conservation biology

## Abstract

Landscape genetics is increasingly being used in landscape planning for biodiversity conservation by assessing habitat connectivity and identifying landscape barriers, using intraspecific genetic data and quantification of landscape heterogeneity to statistically test the link between genetic variation and landscape variability. In this study we used genetic data to understand how landscape features and environmental factors influence demographic connectedness in Europe’s largest brown bear population and to assist in mitigating planned infrastructure development in Romania. Model-based clustering inferred one large and continuous bear population across the Carpathians suggesting that suitable bear habitat has not become sufficiently fragmented to restrict movement of individuals. However, at a finer scale, large rivers, often located alongside large roads with heavy traffic, were found to restrict gene flow significantly, while eastern facing slopes promoted genetic exchange. Since the proposed highway infrastructure development threatens to fragment regions of the Carpathians where brown bears occur, we develop a decision support tool based on models that assess the landscape configuration needed for brown bear conservation using wildlife corridor parameters. Critical brown bear corridors were identified through spatial mapping and connectivity models, which may be negatively influenced by infrastructure development and which therefore require mitigation. We recommend that current and proposed infrastructure developments incorporate these findings into their design and where possible avoid construction measures that may further fragment Romania’s brown bear population or include mitigation measures where alternative routes are not feasible.

## Introduction

Europe is characterised by a fragmented natural landscape, interspersed with a high density of human settlements and associated infrastructure. Habitat loss, fragmentation and infrastructure development are commonly regarded as being among the greatest threats to biodiversity. The construction of highways, has become an issue of increasing concern to wildlife populations^1^. Connectivity models are progressively being used as a tool to address the effects of fragmentation induced by barriers such as highways and other human infrastructure, and landscape resistance models provide a useful method for mapping potential mitigation corridors^2,3^. In a recent study it has been shown that eastern Europe has some of the highest road densities on the continent^4^. However, while 14.7% of the world’s land mass is covered by Protected Areas (PA), globally only 7.5% is covered by connected PAs^5^.

One of Europe’s largest brown bear populations (*Ursus arctos* Linaeus, 1758) is found in the Romanian Carpathians. While the population declined significantly after WWII, when only approximately 800 individuals remained, it recovered to almost 8,000 individuals by 1988^6^. Within the last two decades the population size has been estimated at 6,000 individuals^7^, and the population in the Carpathians has been shown to be one of the most genetically diverse brown bear populations in the world^8,9^. While brown bear habitat has become increasingly fragmented, the species still occupies the same overall distribution as it did during its recent maximum in the 1980’s (Ceausescu period).

Recent developments in road infrastructure are likely to fragment this brown bear population, perhaps for the first time. Faced with this impending threat, bear conservation measures now focus on the maintenance of ecological connectivity. Romanian legislation on ecological corridor assignment (law 57/2007) provides for the protection of connectivity by designating spatially explicit wildlife corridors based on field-informed modelling and empirical validation.

Brown bear distribution is primarily dependent on the availability of food resources. As such, bear home ranges vary widely between different countries and regions^10^. In Scandinavia, male brown bear home ranges vary from 833 to 1,055 km^2^ and from 217 to 280 km^2^ for females in low-density conditions^11^, while in Slovenia male home ranges are estimated at 350 km^2^ ^12^. In Romania brown bears have variable home range sizes and have daily movements within a broad altitudinal range that can vary seasonally^13^. Brown bears exhibit female philopatry, where females live close to or within their mothers’ home ranges^14^ while males disperse over considerable distances^10^. This combination of relatively large home ranges with sex-biased and often long-distance dispersal^15^ makes bears especially susceptible to anthropogenic habitat alteration and barrier effects^16^. Since urban expansion and habitat fragmentation restricts the movement of bears, knowledge of landscape features promoting gene flow is a key factor in the design of wildlife corridors^15^. To better understand the role of landscape features in shaping demographic structure in natural populations, approaches combining genetic analysis with GIS have become increasingly popular^17^. There are a number of emergent tools that can be used to assess landscape connectivity, such as least-cost path or resistance models^18^. Recently, landscape genetics has seen an expansion of new analytical techniques and as such, the usefulness of relatively simple approaches including Mantel^19^ and partial Mantel tests have been debated^20^. Guillot *et al*.^21^ recommended the use of Mantel tests when analysing the independence of two matrices, while alternatives such as multiple regression analysis based on distance matrices (*MRM*) have been successfully implemented in studies regarding the influence of landscape parameters on genetic structure^22–24^. Fine-scale analyses using individual genotype data have been used to determine population processes leading to patterns of genetic structure such as isolation-by-distance (IBD) or landscape resistance^25,26^. Individual genotype data has been used to study fine-scale patterns of genetic variation in bears^27,28^, emphasising the possibility that individual-based approaches in heterogeneous landscapes might be the best method to test landscape connectivity^29^. To infer population genetic structure across the country, and assess genetic distances among individuals we used the same set of microsatellites as in previous studies conducted in Europe including the Carpathians^30^ and southern Europe^31^. Nuclear DNA microsatellites have extensively been used in landscape genetics studies, and have been shown to demonstrate fine-scale resolution, including genetic structure, in these^32,33^ the genetic patterns uncovered by which, have largely been similar to those obtained by whole genome sequencing^32^.

To improve the performance of these methods and to generate a robust modelling framework, relative support (RS) and causal modelling have been implemented to augment the Mantel and partial Mantel test approach^33,34^.

Here we aimed to determine the landscape features that influence gene flow in a heterogeneous environment for brown bears from the Carpathian Mountains by employing a landscape genetics approach, identifying key areas needed for connectivity conservation, and developing a decision tool to assist in management and mitigation strategies. Any proposed infrastructure development will need to consider mitigation measures in areas critical to the health and long-term fitness of brown bears in this region.

## Results

### Genetic diversity and population structure

Null alleles were detected for markers Mu10, Mu15, G10X and Mu09 and these loci were therefore excluded from all subsequent analyses. Deviations from Hardy Weinberg equilibrium were observed for six of the analysed markers, which recorded negative *F*_*IS*_-values that could be attributed to stochasticity, or non-random sampling^35^. Bayesian clustering, employed in STRUCTURE, indicated *K* = 1 (Supplementary material Figs 1 and 3) using LnPr (*X*|*K*) method. Samples were therefore treated as one population and we implemented an individual-based landscape genetics approach.

### Spatial autocorrelation

**Figure 1:**
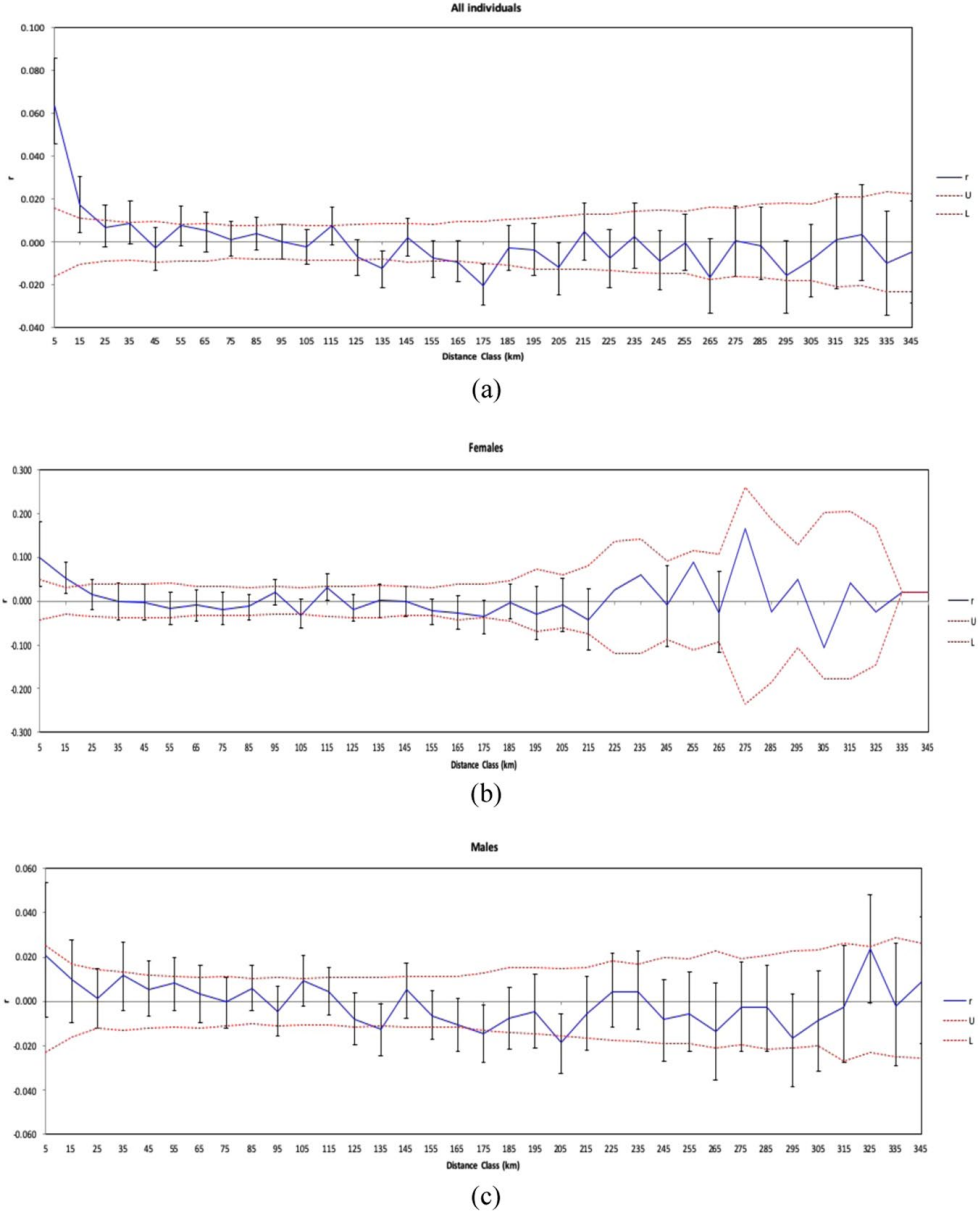
Spatial autocorrelation correlogram of the entire sample (**a**), females (**b**), males (**c**). Two red dotter lines indicate the 95% confidence interval about the null hypothesis of a random distribution of the brown bears. The error bars about r indicate 95% confidence interval determined by bootstrapping. (**a**) All the samples showing a significant and positive autocorrelation for two distance classes (5 km and 15 km). (**b**) Females samples showing a significant and positive autocorrelation values within the first two distance classes (5 km and 15 km). (**c**) Males correlograms indicating not significant values for autocorrelation.

Results showed a higher and significant genetic correlation (class 5 km (*P* = 0.001, r = 0.064) and class 15 km (*P* = 0.003, r = 0.017)) among individuals than expected within specific classes of distance (Fig. 1a). Significant genetic correlation among individuals was detected for females within the first distance class (0–15 km) (class 5 km (P = 0.001, r = 0.10) and class 15 km (P = 0.001, r = 0.051)) (Fig. 1b). For males we detected no significant spatial autocorrelation at any of the distance classes (Fig. 1c).

### Landscape resistance modeling

In order for landscape models to be accepted, models had to pass the following tests: causal modelling with IBR (isolation by resistance) and the reduced model test. In addition, candidate models were also assessed against each other rather than simply against IBR to evaluate models based on relative support (RS). First, we will discuss the results for the best univariate models followed by the best multivariate models.

The best univariate models of effective resistance (Table 1) based on partial Mantel correlations were: rivers (*r* = 0.175; *P* = 0.003), roads (*r* = *0*.091; *P* = 0.016), aspect (*r* = *0*.*076*; *P* = 0.*042*) and slope (*r* = 0.051; *P* = 0.*003*). Large rivers explained about 18% of the variation in genetic distance, followed by roads, eastern aspects and by relatively small slope gradients. Elevation and land use were not significant and were therefore excluded from any further analyses.

**Table 1:** The best univariate models of effective landscape resistances based on partial Mantel correlation after removing the effect of the IBR (isolation-by-resistance) model.

Landscape variable	Parameter values	Partial Mantel *r*	*P*-value
**Rivers** (**riv2**)	Classified; R_max_ = 2	***0***.***175***	***0***.***0003***
**Roads** (**ro2**)	Classified; R_max_ = 2	***0***.***091***	***0***.***016***
**Aspect** (**a71**)	90°; *x* = 10; *R*_max_ = 2	***0***.***076***	***0***.***042***
**Slope** (**sl68**)	15°; *R*_max_ = 100	***0***.***051***	***0***.***0033***
RoadLoc (rl2)	Classified; R_max_ = 2	*0*.*067*	*0*.*058*
Elevation (de4)	500; *R*_max_ = 100	*0*.*052*	*0*.*094*
Land Use (clc26)	Forest cover; *R*_max_ = 100	*0*.*011*	*0*.*385*

Best-supported model is ranked at the top, we reported optimized parameter values, partial Mantel *r* and significance of support. In bold are indicated the supported models.

Causal modelling with IBR for rivers was defined as such: partial Mantel test between genetic distance and rivers after removing the effect of IBR was *significant* (*P* = 0.002) and partial Mantel test between genetic distance and IBR, removing the effect of the rivers was *not significant* (*P* = 0.997), suggesting that landscape resistance as a function of rivers was supported (Table 2).

**Table 2:** The best univariate models of landscape resistance based on relative support (RS) and causal modelling.

Landscape variable	Parameter values	RS_IBR_	(*1*) *r*	(*1*) *P*	(*2*) *r*	(*2*) *P*	Supported
**Rivers** (**riv1000**)	Classified; *R*_max_ = 1000	***0***.***274***	***0***.***143***	***0***.***002***	**−*0***.***131***	***0***.***997***	**Yes**
**Aspect** (**a71**)	90°; *x* = 10; *R*_max_ = 2	***0***.***075***	***0***.***076***	***0***.***042***	***0***.***0007***	***0***.***500***	**Yes**
**Roads** (**ro10**)	Classified; *R*_max_ = 10	***0***.***074***	***0***.***073***	***0***.***042***	**−*0***.***0007***	***0***.***505***	**Yes**
RoaLoc (rl2)	Classified; *R*_max_ = 2	*0*.*011*	*0*.*067*	*0*.*058*	−*0*.*056*	*0*.*913*	No
Slope (sl68)	15°; *R*_max_ = 100	−*0*.*051*	*0*.*051*	*0*.*0033*	*0*.*102*	*0*.*0003*	No

Optimized parameter values, RS as compared to IBR, partial Mantel *r* and significance of support are reported. Optimized values include equation parameters for *x* or SD (contrast; shape of the relationship) and *R*_max_ (magnitude of the relationship). (1) partial Mantel test between genetic distance and the landscape variable, partialling out the effect of IBR (GD~LV|IBR); (2) partial Mantel test between genetic distance and IBR distance, removing the effect of the landscape variable (GD ~ IBR|LV). Mantel *r*-value is reported in the first column of each test while in the second column we reported *P*-value. We indicated supported models in bold.

Model optimisation based on RS, showed that the best univariate model was rivers when compared to the IBR model, followed by aspect and roads (Table 2). Only rivers, aspect and roads have met the IBR causal modelling criteria. R_max_ values were greater for rivers and roads than in previous univariate models using casual modelling, while model parameters for aspect remained constant.

Causal modelling after removing the effect of IBR calculated for Ri + Ro + A (Table 3; Ri – rivers, Ro – roads, A – aspect) shown that: (1) partial Mantel test between the genetic distance and Ri + Ro + A after removing the effect of IBR (GD ~ (Ri + Ro + A)|IBR *was significant* (*P* = 0.037, Table 3; column 1); (2) partial Mantel test between genetic distance and IBR partialling out the landscape variable (GD ~ IBR|(Ri + Ro + A) *was not significant* (*P* = 0.671, Table 3; column 2); (3) the partial Mantel correlation *was significant* (*P* = 0.003, Table 3; column 3) when partialling out the effect of the reduced model (GD ~ (Ri + Ro + A)|(Ri + Ro); 4) while the opposite (GD~(Ri + Ro)|(Ri + Ro + A) was *not significant* (*P* = 0.959, Table 3; column 4). Model optimization based on RS, indicated that the best multivariate model comprised of aspect and rivers (Table 3, model 1). Relative support was calculated with the following formula:

$$R{S}_{1|2}=(A)-(B)$$

where (A) was the partial Mantel test between genetic distance and landscape variable 1 (LV_1_), partialling out the effect of second landscape variable (LV_2_) and (B) was the partial Mantel test between genetic distance and LV_2_ partialling out the effect of LV_1_. In order to be supported, RS of the first model compared to the second model should be positive in every comparison. Including aspect in multivariate model 1 did not improve the RS value (0.097). The RS for model 2 compared to model 1 was slightly lower, but this model still passed the causal modeling criteria. This model included the same parameters as in the univariate analysis (Table 1). In addition, model Ri + Ro + A performed significantly better (*r* = 0.079, *P* = 0.037) than resistance distance alone (*r* = −0.018, *P* = 0.671).

**Table 3:** The best multivariate models based on relative support (RS), causal modelling after removing the effect of the isolation-by-resistance (IBR) model (1, 2) and causal modelling criteria with the reduced model (3, 4).

	Model	Parameters	RS_IBR_	(1) *r*	(1) *P*	(2) *r*	(2) *P*	(3) *r*	(3) *P*	(4) *r*	(4) *P*
(1)	Ri + Ro	*Ri*: *Classified*; *R*_max_ = 2	*0*.*162*	*0*.*088*	*0*.*019*	−*0*.*074*	*0*.*964*	*Ri*: *0*.*039*	*0*.*182*	*Ri*: −*0*.*024*	*0*.*705*
		*Ro*: *Classified*; *R*_max_ = 2						*Ro*: −*0*.*044*	***0***.***843***	*Ro*: *0*.*058*	***0***.***088***
(2)	Ri + Ro + A	*Ri*: *Classified*; *R*_max_ = 2	*0*.*097*	*0*.*079*	*0*.*037*	*−0*.*018*	*0*.*671*	***Ri***: **−*0***.***008***	***0***.***012***	***Ri***: ***0***.***018***	***0***.***855***
		*Ro*: *Classified*; *R*_max_ = 2						*Ro*: *0*.*009*	*0*.*417*	*Ro*: *0*.*038*	*0*.*183*
		*A*: 90°; *x* = 10; *R*_max_ = 2						***A***: **−*0***.***074***	***0***.***003***	***A***: ***0***.***113***	***0***.***959***

We reported optimized parameter values, RS as compared to IBR, partial Mantel r and significance of support. Optimized values include equation parameters for x or SD (contrast; shape of the relationship) and R_max_ (magnitude of the relationship). Causal modeling after removing the effect of the IBR consisted in: partial Mantel test between genetic distance and the landscape variable, partialling out the effect of IBR (GD ~ LV|IBR) and partial Mantel test between genetic distance and IBR, removing the effect of the landscape variable (GD ~ IBR|LV). Causal modeling criteria with the reduced model was: (3) partial Mantel test between genetic distance and landscape model after removing the effect of the reduced model GD ~ LM|) and partial Mantel test between genetic distance and the reduced model, partialling out the effects of the landscape model (G ~ |LM). Mantel r-value is reported in the first column for each test while the second column is reported the P-value. Abbreviation: Ri – River, Ro – Road, A – Aspect.

The best multivariate model based on the partial Mantel correlation after partialling out the IBR model (Table 3, column 1: *P* = 0.019; column 2: *P* = 0.964) included rivers and roads (model 1). Roads, rivers and aspect (model 2) were supported based on the partial Mantel correlation after removing the effect of IBR (Table 3).

The best-supported model using the AICc statistic consisted of a combination of aspect, rivers, roads and slope (C: *R*^2^ = 0.021, w_i_ = 1). When VIF values were calculated we observed collinearity between roads and rivers. We therefore calculated MRM excluding rivers (model C1; *R*^2^ = 0.020, w_i_ = 1; Table 4). When we excluded roads, model C_2_ showed a lower AICc value (Table 4). Rivers, aspect and slope were identified as the landscape predictors influencing gene flow.

**Table 4:** Multiple regressions on distance matrices (MRM) indicating the relationship between pairwise genetic distances and the resistance distances for different landscape variables.

Model Name	Variables	β	*P*	R^2^	*P*	VIF	AICc	∆AICc	Weight (*w*_*i*_)	RI
				0.0530	0.0010		36300.3	1.27	0.625	5.31
**A**	Aspect		*0*.*102*			2.97				
	Rivers		*0*.*001*			***782***.***65***				
	RoadLoc		*0*.*850*			***172***.***16***				
	Roads		*0*.*170*			***38***.***76***				
	Slope		*0*.*613*			3.76				
	IBR		*0*.*001*			**858**.**97**				
**Excluding IBR**				0.0229	0.0180		35714.5	4.05	0.883	2.3
**B**	Aspect		*0*.*105*			2.97				
	Rivers		*0*.*417*			***103***.***03***				
	RoadLoc		*0*.*140*			***159***.***63***				
	Roads		*0*.*230*			***38***.***60***				
	Slope		*0*.*421*			3.75				
**Excluding RoadLoc**				0.0211	0.0130		35682.2	0.00	1	2.12
**C**	Aspect		*0*.*012*			2.96				
	Rivers		*0*.*131*			***28***.***61***				
	Roads		*0*.*235*			***24***.***69***				
	Slope		*0*.*228*			3.68				
**Excluding Rivers**				0.0200	0.0080		35661.5	0.00	1	2
**C1**	Aspect		*0*.*013*			2.90				
	Roads		*0*.*023*			4.13				
	Slope		*0*.*022*			3.29				
**Excluding Roads**				**0**.**0183**	**0**.**0120**		**35630**.**2**	**0**.**00**	**1**	**1**.**83**
**C2**	**Aspect**	***0***.***1253***	***0***.***002***			**2**.**94**				
	**Rivers**	***0***.***0748***	***0***.***000***			**4**.**79**				
	**Slope**	−***0***.***0770***	***0***.***000***			**3**.**66**				

In the model C1 and C2 we alternately removed roads and rivers in order to minimize colinearity among predictors. We reported β = intercept only for the best-supported model while *P* = *P*-value and VIF = Variance Inflation Factor were reported for each model. We present the results of matrix regressions (model R^2^) and Akaike’s Information Criterion (AICc, ∆AICc, *w*_*i*_). Models with the highest AIC support are in bold (∆AICc ≤ 2).

### Current density maps and connectivity

**Figure 2:**
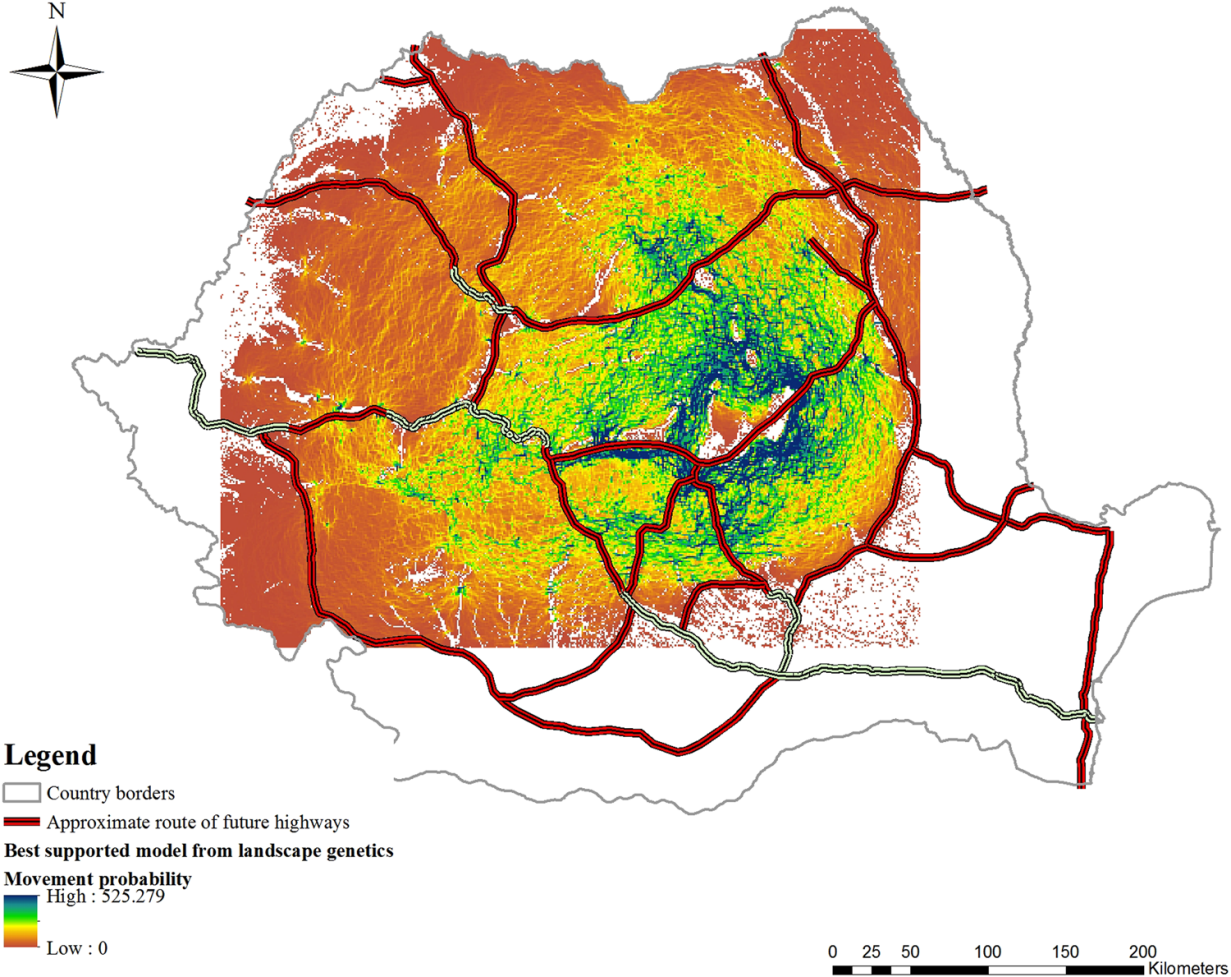
Best-supported map of the landscape parameters that are influencing gene flow, existing highways (green) and future developing infrastructure layer (red). Blue and green cells (1 km × 1 km) represents the highest probability for movement, while orange cells (1 km × 1 km) represents a lower probability for brown bear movement paths.

**Figure 3:**
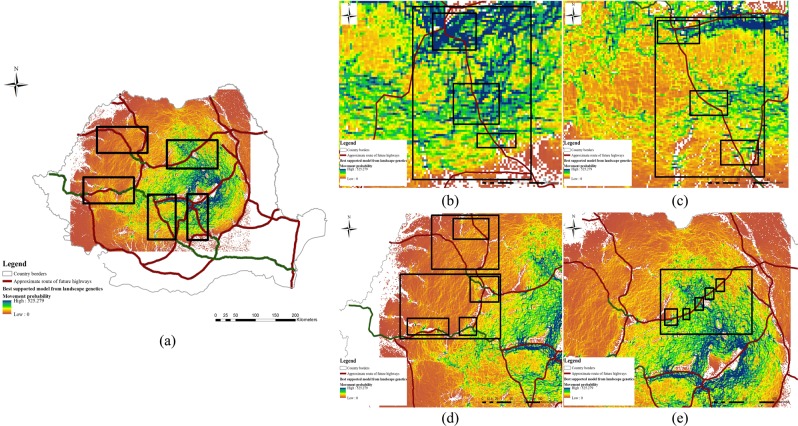
Case studies location general view (**a**,**b**) Prahova Valley, (**c**) Olt Valley, (**d**) Apuseni, (**e**) Targu Mures – Iasi. For all the maps blue and green cells (1km x1km) represents the highest probability for movement, while orange cells (1 km × 1 km) represents a lower probability for brown bear movement paths. (**b**) Prahova Valley: two major and one small wildlife corridors (black rectangles) for each of the areas. The existing roads already exercise pressure on species movement due to very high traffic intensity and the topography of the valley. (**c**) Olt Valley: one major and two small wildlife corridors (black rectangles) for each of the areas. The existing roads already exercise pressure on species movement due to very high traffic intensity and the topography of the valley. (**d**) Apuseni: a network of small wildlife corridors within four rectangles (black colour) which are grouped in two large rectangles (black colour) at regional levels. (**e**) Targu Mures – Iasi: a network of three small corridors and two large ones grouped in a regional rectangle (black colour).

**Figure 4:**
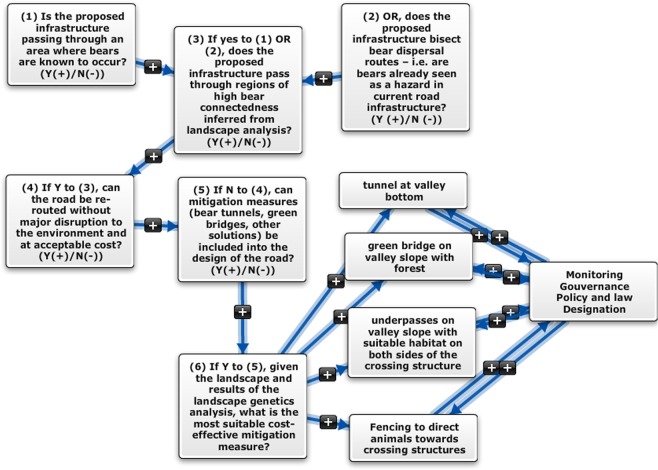
Decision tool for identifying the most suitable measures and cost effective for mitigating the impact of new highway development based on the landscape genetics models. The steps to be follow are meant to ease decision-making and to identify cost effective measures for gene flow to thrive.

Using the best multivariate model, a map highlighting core areas with the highest current density (areas of connectedness) for brown bears along the Carpathians was generated. The current map of the most probable routes of gene flow indicated the presence of wildlife corridors which plays a major role in maintaining gene flow within this large brown bear population (Fig. 2). We therefore identified several wildlife corridors in areas were development has been planned (Fig. 3a): Prahova Valley (Fig. 3b, 3 corridors), Olt Valley (Fig. 3c, 3 corridors), Apuseni (Fig. 3d, 3 large areas with small corridors) and Targu Mures – Iasi (Fig. 3e, 5 corridors). Some of the corridors are large, while others are very small. However, they are not the only ones, there are others that have not been the subject of this study. Moreover, we generated a framework of decision (Fig. 4) for new highways to be built based on our landscape genetic model outputs: (a) identifying if the infrastructure passing through an area where bears are known to occur; (b) identifying if the proposed infrastructure bisect bear dispersal routes; (c) inferring if the proposed infrastructure pass through regions of high bear connectedness as a result of the landscape analysis; (d) analysing if the road can be re-routed without major disruption to the environment and at acceptable cost; (e) identifying mitigation measures (bear tunnels, green bridges, other solutions) which can be included into highway design; (f) given the landscape and results of the landscape genetics analysis, identifying the most suitable and cost-effective mitigation measure (bear tunnel at valley bottom, green bridge on valley slope with forest, etc.).

## Discussion

Model-based clustering inferred one large population; the overall lack of genetic sub-structure suggests high levels of dispersal and gene flow. This implies that bear habitat has yet to become fragmented to restrict movement of individuals. This is also the case in nearby Croatia where a single population for brown bears was detected and highlighted the importance of habitat connectivity^36^. Spatial autocorrelation revealed a significant genetic correlation among females at relatively fine spatial scales (up to 14 km) supporting observations of female philopatric behaviour^14^. A previous study^37^ showed that females can disperse up to 10 km while males disperse on average over distances of 30 km. These observations are supported by telemetry research carried out at the National Institute for Research and Development in Forestry Marin Dracea (INCDS Marin Dracea) in order to establish home ranges of brown bears in Romania (10 to 25 km^2^ for females). The pattern detected of fine-scale spatial correlation in females could be explained by young females establishing their home ranges close to their mothers^14^, although we did not have information on the age of the approximately 50 females analysed here. As expected, males did not show significant spatial correlation, confirming ecological evidence for long-distance juvenile male dispersal^38^.

The optimal landscape model predicting genetic connectivity among individuals comprised a combination of rivers and eastern facing slopes. Large rivers, often located alongside villages and large roads featuring heavy traffic, were found to restrict gene flow, while eastern facing slopes promoted dispersal. These results are surprisingly similar as to those recently found for giant panda in the Qinling mountains in China, and could be linked to abundant vegetation and food resources^39^. In our study, land cover did not significantly correlate with gene flow. This is likely to be due to a lack of fragmentation of forest regions at a national level in Romania^40^. Similarly, other studies have reported a lack of correlation with land cover^34^, thus we infer that bear preferences for slope and specifically eastern facing slopes could be determined by forest type and food availability (as with the giant panda)^41^. A recent study carried out in the eastern Romanian Carpathians using telemetry showed that during hyperphagia bears seeking food were capable of moving along altitudinal gradients of high variability^42^, moreover, when analysing an extensive telemetry dataset of 70964 GPS locations collected from 32 brown bears (from which 9 were males) across Romania (INCDS Marin Dracea research projects, unpublished results) a high preference for the forest (especially mixed Norway spruce-silver-fir-beech and beech dominant forest) was revealed (Figs S5 and S6). In Romania, European beech and Norway spruce are found in large stands and high intrapatch/interpatch connectivity has been observed^40^ suggesting that bears benefit from these favorable conditions in terms of food availability, shelter and movement. Slope aspect in beech forests seems to be an extremely important for herbaceous composition: a lower number of species but with more mature vegetation are present on north-eastern aspects from April to August, suggesting that north-eastern slopes could have higher biomass production due to more humid soil and competition for light^43^, facilitating thus bear movement in accordance with food availability. Moreover, Ciucci *et al*.^44^ described the preference of the brown bears for consumption of herbaceous vegetation in spring and early summer, berries in latte summer and hard mast in the autumn (periodically available from beech (*Fagus sylvatica*), oak (*Quercus*) and common hazel (*Corylus avellana*)).

In our study, bears were inferred to use the gentlest slopes available and this likely reflects the energetic cost of dispersal^45^. However, topographic variables were not significant in a previous, broad-scale habitat model for brown bears across Poland^46^, while studies in other parts of Europe^47–49^ implied a preference for rugged terrain. This observation might be explained by the geography of the Romanian Carpathian Mountain range, which is of low to medium altitude and no wider than 100 km, deeply fragmented by longitudinal and transverse valleys crossed by several major rivers; surrounded by large ground cavities. These features are expected to facilitate the movement of brown bear individuals by selecting the easiest paths due to energetic constraints.

When superimposed on the future infrastructure map, our landscape model suggested the presence of wildlife corridors in important areas for connectivity (Fig. 3a): (1) the Prahova Valley (Fig. 3b) also features a European-level road with extremely heavy traffic and a railway, crossing one of the most popular tourist destinations in the country (Sinaia, Busteni, Predeal, Azuga); (2) the gorge of the Olt River which includes many artificial lakes, and features extremely heavy traffic and a railway line (Olt Valley, Fig. 3c); while 3) Apuseni (Fig. 3d) and (4) Targu Mures – Iasi (Fig. 3e) are included in the future development plan. The influence of European roads/highways is likely to be considerable, especially due to their collinearity with large rivers, and they are likely to have become more and more restrictive due to traffic intensification during the last two decades. However, highways with properly designed mitigation measures can ensure connectivity despite roads/railways/river systems.

Romanian bear habitat remains among the least fragmented in Europe (Joint EEA-FOEN report). If current traffic density plays an important role in restricting dispersal and gene flow in brown bears and road/railway mortalities confirms this (see Fig. S7), the proposed extension of the developing transport network could have a substantial impact on brown bears, which are not currently affected by dispersal constraints. Over the next few years, governmental road infrastructure development will include the construction of highways crossing core brown bear habitat and this is likely to cause further fragmentation if connectivity is not ensured. Since anthropogenic fragmentation acts rapidly and can affect the adaptive potential of populations^50^, investigating its effects *post hoc* can be obscured by time lags in response to disturbance^51^. However, landscape resistance models are a useful tool in mapping potential corridors and predicting connectivity prior to the construction process^52^, as we have attempted to apply here.

While economic development is seen as critical for any country, we advocate the use of landscape analyses to drive ‘smart’ development, by suggesting optimal mitigation measures. Restoration of wildlife habitats and ecosystem functionality is far more expensive than applying measures in advance to reduce fragmentation risks.

At a national level, landscape modelling can act as a decision-support tool for stakeholders to inform connectivity management and to encourage evidence-based conservation. Fine-scale maps of landscape resistance can be used to counteract the potential effects of habitat fragmentation and anthropogenic disturbance. Our approach can be seen as a means to inform authorities about ongoing ecological changes and could encourage a proactive approach to the design of infrastructure development through the proposed decision tool described in Fig. 4.

Areas where wildlife corridors might have the highest benefits for bear connectivity should be prioritised (Prahova Valley (Fig. 3b), Olt Valley (Fig. 3c), Apuseni (Fig. 3d) and Targu Mures-Iasi (Fig. 3d)) and barrier mitigation measures should be considered for improving connectivity e.g. considering these particular cases: (1) when European/national roads have very high volumes of traffic (while no highway is planned to be developed in the area) and wildlife corridors are identified and still used by brown bears, local measures should be applied: e.g. signs for reducing speed, warnings to drivers, improving driver visibility, installing an audible system to alarm wildlife when they are approaching a road; and (2) when a highway/railway is due to be constructed, mitigation measures such as overpasses and underpasses should be considered from the early planning stages (Fig. 4). Such an approach will reduce costs compared to when measures are adopted at later stages. While our results are robust with respect to recent changes in the landscape, fragmentation effects can be biased by time lags in species response, and corridor mitigation should be considered when implementing future developments. Brown bear conservation will depend on the management of corridors, monitoring of wildlife populations, evidence-based science outputs, collaborations between scientists, stakeholders and policy makers, and clear policies on landscape connectivity for wildlife species.

Furthermore, ensuring connectivity would have multiple benefits not only for biodiversity but also for other environmental objectives, while avoiding unnecessary or potentially bureaucratic mitigation planning. With these connectivity measures in place that will promote gene flow in the Carpathians, brown bear has a unique chance to co-exist with economic development.

## Materials and Methods

### Study area, sample location and genotyping

The study was carried out in the Romanian Carpathians, covering an area of approximately 69,000 km^2^ (Supplementary Material; Fig. S4). A total of 199 tissue samples were collected (Supplementary Material; Fig. S4) over three years under the annual minimum level of interventions for derogation. DNA was extracted from tissue samples preserved in 99% ethanol using the Maxwell®16 Tissue DNA Purification Kit (Promega, USA) following an optimised protocol^53^. Seventeen fluoro-labelled microsatellite markers^54–57^ were used, divided into four multiplexes: Multiplex I (Mu50, Mu59, Mu09, Mu10, Mu15), Multiplex II (G10B, G10L, G1A, Mu51), Multiplex III (G10J, G10M, Mu61, G10X), Multiplex IV (G10C, G10D, G10P) and a singleplex reaction (SRY). Amplification was performed in 15 μl containing 7.5 μl of the Qiagen Multiplex PCR mix, 50–100 ng/ul DNA and 0.2 and 0.4 μM of forward and reverse primers respectively. Fragment analysis was performed using a GenomeLab™ GeXP Genetic Analysis System.

### Statistical analysis

Microsatellite data was tested for the presence of null alleles using MICROCHECKER^58^. Observed and expected heterozygosity was estimated for each locus using GENETIX 4.05^59^, while Hardy-Weinberg equilibrium was analysed for each locus using GENEPOP v.4.2^60^. Statistical significance was assessed by *P*-values using a sequential Bonferroni correction for multiple comparisons^61^.

We used a Bayesian clustering method in STRUCTURE v 2.3.4^62,63^ to infer population structure using 1,000,000 simulations preceded by 100,000 burn-in replicates assuming an admixture model and correlated alleles frequencies. *K* values varied from one to ten and we performed ten repetitions for each *K* value. Both the posterior probability of the data for the given value of *K* (Ln Pr(X|K)) and its rate of change (Δ*K*) were used to identify the number of population clusters^64^. We generated genetic distances between individuals based on the proportion of shared alleles (*Dps*) in MSA^65^. We estimated pairwise Euclidean geographic distances between all sampling points allowing to test for the presence of a simple (isolation-by-distance) IBD^66^ model, which assumes genetic differentiation is a by-product of geographic distance without taking any landscape features into account. IBD was calculated in GenAlEx v 6.5^67^ using 10,000 random permutations. We investigated the genetic autocorrelation of multilocus genotypes at multiple spatial scales using GenAlEx v 6.5^67^. We then performed spatial autocorrelation, in addition, spatial autocorrelation was examined within males and females to test for sex-specific differences in autocorrelation. The 95% confidence interval around the point estimate of correlation (r) was obtained by bootstrapping with replacement. A value of r was considered statistically significant if its 95% confidence interval was above the null hypothesis of r = 0. We also performed multiple regressions on distance matrices *MRM* (R; “ecodist” package)^68^ and this method was used to identify the contribution of each landscape variable to the overall variance in the dependent variable (genetic distance), since analysing data using only one method could result in false, method-dependent outcomes^69,70^. AICc weights (*wi*) were calculated by using the “MuMIn” R package^71^. Second-order Akaike Information Criterion values (AICc) were calculated among all competing models based on resistance distances, in order to select the best model (smallest AICc)^72^. Models that registered the lowest change in the AICc score (∆AICc = 0) and the highest Akaike weight were considered optimal^73^. To test for collinearity, the Variance Inflation Factor (VIF) was calculated for each predictor^74^ in the model using the “VIF” R package. The presence of multi-collinearity, VIF values >10^75^ revealed a linear association between two explanatory variables. Beta values (β) was calculated for the best model, in order to detect which of the independent variables had a greater effect on the dependent variable^76^ using the R package “QuantPsyc^77^”. Raster maps for aspect, elevation, and slope were extracted from a Digital Elevation Model (DEM) at a resolution of 1 km square pixels. In addition, land cover (CLC 2012), rivers and roads maps with the same resolution were used. In order to assign resistance values to each cell the “Reclass” function^78^ in ArcGIS v 10.1 (ESRI 2012) was used and rasters were converted to ASCII files to be used in CIRCUITSCAPE v.3.5^79^. The landscape parameters considered in this study are listed in Table S2 (Supplementary Materials).

### Landscape resistance

An isolation-by-resistance (IBR) raster was generated in CIRCUITSCAPE v.3.5 by assigning a value of 1 to all pixels. Resistance distances between individuals were generated in CIRCUITSCAPE v.3.5, using the pairwise modelling mode with focal points and connected to eight neighbours^80^. We determined the best univariate models of effective landscape resistances based on partial Mantel correlation after removing the effect of the IBR model using the “vegan” R package^81^. In addition, a causal modelling approaches were used^82–84^ that consisted of two steps: the first step suggests that if a resistance hypothesis model is supported independently of the null model then partial Mantel tests between the genetic distance and the environmental variable would be significant after removing the effect of IBR; partial Mantel tests between genetic distance and IBR distance would not be significant, partialling out the environmental variable. The second step allows for the comparison of causal modelling with a reduced model. If the true model is supported independently of the other candidate models then partial Mantel tests between genetic distance and the true model would be significant, removing the effect of the reduced model and partial Mantel tests between genetic distance and the reduced model would not be significant, partialling out the effect of the true model.

Scaled transformations^85^ for each landscape variable were ranked based on partial Mantel correlation coefficients after removing the effect of IBR using 9,999 permutations^19^. The function with the highest partial Mantel *r*-value (significant *P*-value) was included in the next step of the analyses. Further, the best univariate models based on relative support (RS) and causal modelling were evaluated after removing the effect of IBR, thus we tested if landscape resistance was supported independently by the null model^86^.

Models were ranked by model support values. Optimized values included parameter equations for *x* or SD (contrast and the shape of the relationship, respectively) and *R*_max_ (magnitude of the relationship). The first step included a partial Mantel test between genetic distance and the landscape variable (LV), partialling out the effect of IBR (A = GD ~ LV|IBR); while the second step included a partial Mantel test between genetic distance and the IBR model, removing the effect of the landscape variable (B = GD ~ IBR|LV). In order for a landscape variable to be included in the final multivariate model, it had to pass the causal modeling criteria with the reduced model. We report optimized parameter values, RS compared to IBR, partial Mantel *r*-values and significance of support.

Multivariate resistance surfaces were built by generating rasters equal to the sum of univariate model rasters for each landscape feature. First models were built using the best two landscape variables (rivers and roads, highest partial Mantel *r*) by maintaining parameters of the first variable constant, while the second variable parameters were varying. We identified the best-supported model for the second variable by analysing the partial Mantel correlation (after removing IBR). The optimum parameter of the first variable was obtained by keeping the parameter of the second variable constant. Additional landscape variables (aspect and slope) were added one at a time by keeping the first two constant until the best-supported model did not change. We also evaluated multivariate models by their RS rather than just removing the effect of IBR. Both procedures were repeated until all parameters were stable.

Cumulative current maps were generated in CIRCUITSCAPE v.3.5, best-supported maps of the landscape parameters that influenced gene flow have been generated by combining the cumulative currents maps in ArcGIS, displaying the road infrastructure layer and the current infrastructure development plan.

## Supplementary information

Supplementary Fedorca et al 2018 Nature Scientific Reports.docx

## Data Availability

Distance (genetic and resistance) matrices; raw microsatellite data and GIS layers will be archived on DRYAD.
