# Supplementary material for: Inferring fine-scale spatial structure of the brown bear (Ursus arctos) population in the Carpathians prior to infrastructure development
Source: Sci Rep. 2019 Jul 1;9:9494. doi: 10.1038/s41598-019-45999-y (PMC6602936; doi:10.1038/s41598-019-45999-y)
Supplement: Supplementary file 1 — Supplementary Fedorca et al 2018 Nature Scientific Reports.docx [file 41598_2019_45999_MOESM1_ESM.docx]

**Inferring fine-scale spatial structure of the brown bear (Ursus arctos) population in the Carpathians prior to infrastructure development**

**Ancuta Fedorca^1,2*$^, Isa-Rita M. Russo^3*^, Ovidiu Ionescu^1,2^, Georgeta Ionescu^1,2^, Marius Popa^1,2^, Mihai Fedorca^1,2^, Alexandru Lucian Curtu^2^, Neculae Sofletea^2^, Gary M. Tabor^4^ and Michael W. Bruford^3^**

**Table S1** Basic genetic parameters for the entire sample

| **Locus** | **Na** | **Ne** | **Ho** | **He** |
| --- | --- | --- | --- | --- |
| **G10J** | 13 | 3.742 | 0.779 | 0.733 |
| **MU50** | **17** | 6.203 | 0.864 | 0.838 |
| **G10C** | 13 | 6.352 | 0.889 | 0.842 |
| **MU59** | **17** | 8.350 | 0.884 | 0.880 |
| **MU10** | 12 | 5.290 | 0.738 | 0.811 |
| **MU51** | 9 | 4.640 | 0.819 | 0.784 |
| **G10B** | 13 | 3.908 | 0.834 | 0.744 |
| **MU61** | 9 | 4.370 | 0.778 | 0.771 |
| **G10L** | 14 | 8.901 | 0.869 | 0.887 |
| **G10D** | 9 | 4.255 | 0.824 | 0.765 |
| **G1A** | 10 | 5.363 | 0.924 | 0.813 |
| **G10M** | 9 | 3.254 | 0.733 | 0.692 |
| **Mean** | **12** | **5.386** | **0.828** | **0.797** |

Na – number of alleles; Ne – effective number of alleles;

Ho - Observed heterozygosity; He - Expected heterozygosity

*Equation S1 Elevation – Inverse Gaussian function^1^*

R = R_max_ + 1 - R_max_ * e^-(Elevation – Elevation^_opt_^)2^/^(2*(E2^_SD_^)^

𝑅_𝑚𝑎𝑥_ - maximum resistance

𝐸_𝑜𝑝𝑡_ - optimal elevation

𝐸_𝑆𝐷_ - standard deviation about the optimal elevation

Parameter values tested were: 𝑅_𝑚𝑎𝑥_= 2, 10, 100, 500, and 1000; 𝐸_𝑜𝑝𝑡_= 500, 1000, 1500, 2000 m; 𝐸_𝑆𝐷_= 100, 200, 300 m for a total of 60 candidate models.

*Equation S2 Aspect – Inverse Gaussian function^1^*

R=[(1-cos (θ- θ_opt_))/2]^x^_*_R_max_ +1

𝑅_𝑚𝑎𝑥_ - maximum resistance

θ _𝑜𝑝𝑡_ – hypothesized optimal aspect, resistance increases toward R_max_ (θ _𝑜𝑝𝑡_+180**°**)

Parameter values tested were: 𝑅_𝑚𝑎𝑥_= 2, 10, 100, 500, and 1000 by using five exponential variables x = 0.5, 1, 2, 4, 10, was modelled by adding 45**°** increments from 0**°** to 315**°**, for a total of 200 candidate models

*Equation S3 Slope – Inverse Gaussian function^1^*

R = R_max_ + 1 - R_max_ * e^-(Slope – Slope^_opt_^)2^/^(2*(E2^_SD_^)^

𝑅_𝑚𝑎𝑥_ - maximum resistance

S_𝑜𝑝𝑡_ - optimal slope

S_𝑆𝐷_ - standard deviation about the optimal slope

Parameter values tested were: 𝑅_𝑚𝑎𝑥_= 2, 10, 100, 500, and 1000; S_𝑜𝑝𝑡_= 5, 10, 15, 20, 25, 30; S_𝑆𝐷_= 100, 200, 300 m for a total of 60 candidate models.

*Land cover categorization*

We reclassified a land cover raster into four categories

1.mixed forests

2.deciduous

3.coniferous forests

4.other anthropic areas (mines, railroads, cities, villages, urbanized areas, industrial areas)

We ranked each cover class by giving the lowest rank for mixed forests followed by deciduous, coniferous forests and other anthropic areas. We reclassified the resulting categorical rank raster according to the function R = Rank^x^.

We tested five values of x and six values of R_max_ (2, 10, 100, 250, 500 and 1000), for a total of 45 candidate models.

Causal modelling description

*IBR – Isolation-by-resistance*

*LV – Landscape variable*

*GD – Genetic distance*

*LM – Landscape model*

Table 2 Landscape parameters considered in landscape scenario

| **Variable** | **Description** | **Justification** | **Classification** |
| --- | --- | --- | --- |
| **Land cover**  **Land use** | Land cover was modelled by assuming that recorded suitable habitats provide less resistance than unrecorded habitat by using four categories:   - Percentage of mixed forests (as movement facilitator); - Percentage of deciduous forests; - Percentage of conifer forests; - Percentage of anthropogenically altered areas | Brown bears are highly dependent on large landscapes and forest cover, mating occurs in highly specialized habitats^2^. According to Moe et al. (2007)^3^ forest was an important determinant of brown bear habitat, while Ziolkowska et al. (2016)^4^ showed that bears selected mixed forests over forests with a high share of deciduous trees. Likewise, Fernandez et al. (2012)^5^ indicated that deciduous forests with hard mast tree species are important for bears. | We reclassed resistances for land cover according to Castillo *et al.* (2014)^1^ and we evaluated the same R_max_ values for a total of 45 candidate models. |
| **Water bodies** | Water bodies were modelled by using a simple water-land dichotomy. We modelled resistance as a function of water bodies considering only large water bodies (large rivers, lakes, artificial lakes) as restricting gene flow. Small rivers and streams were considered not to restrict bear movements. | It has been shown that roads and rivers act as barriers to movement and restrict gene flow^6^. Permeability is correlated with the ecological cost of movement, accordingly it is more likely that organisms experience the landscape matrix as a gradient of differential permeability^7^. Thus, the combined effect of roads and rivers can act as barriers increasing the brown bears movement costs. | A value of 1 to land, while for big rivers we assigned like above series of different maximum resistances (R_max_). |
| **Aspect** | Aspect was modelled by adding 45**°** increments from 0**°** to 315**°.** | McCune & Keon (2002)^8^ proposed that northeast aspects represent cooler slopes, while the southwest aspects are warmer. Few studies: Spear & Storfer (2008)^9^, Castillo et al. (2014) ^1^, Russo et al. (2016)^10^ have found that eastern and nord-eastern aspect represents gene flow predictors in small mammal species, especially due to preference for various types of habitats found on those aspects^11^. For satisfying food requirements bears are dependent on both plants and animals, likewise tree seed and forest fruits, these facilitating individual’s movements^12^. However, aspect can represent a potential answer for seasonal movements^5^ | We hypothesised that for the Carpathians northeastern is the optimal aspect such that bears will select this aspect due to habitat preferences and food availability We evaluated five maximum resistance (R_max_) values (2, 10, 100, 500 and 1000) and five exponential variables (0.5, 1, 2, 4, 10) for a total of 200 candidate models. Aspect was modelled using the equation provided in Castillo *et al.* 2014 (Supplementary material)^13^ |
| **Slope** | We hypothesized that bears minimize energetic costs when moving, selecting small to moderate slopes. | Carnivores are some of the widest ranging terrestrial mammals for their size, and this affects overall energy budgets^14^. In a previous study done by Cushman *et al.* (2006)^15^ the gene flow in a black bear population was facilitated by forest cover at middle elevations and not influenced by slope. However, slope and elevation are the most informative measures for movement^16^. In various studies bears has selected for moving small to medium slopes^5,17-19^ | Slope was reclassified according to an inverse Gaussian function (Supplementary materials) used by Castillo *et al.* (2014) and we evaluated five maximum resistance (R_max_) values (see above), six optimum slopes (5, 10, 15, 20, 25, 30) and three standard deviations for a total of 90 candidate models. |
| **Elevation** | We hypothesized that bears minimize energetic costs when moving, selecting medium elevation. | Territorial conditions can be very different to those facilitating dispersal, thus it is important to understand how the environment influences animals during movements such as mating excursions^20^. In a previous study done by Cushman *et al.* (2006)^15^ the gene flow was facilitated by forest cover at middle elevations. | We reclassified digital elevation the same as slope and we evaluated five maximum resistance (R_max_) values (see above), four optimum elevations (500, 1000, 1500, 2000) and three standard deviations for a total of 60 candidate models. |
| **Roads** | Roads were classified in the same way but here we used a European road/highway vs no-road (plus other smaller categories) dichotomy. In addition, we hypothesised that areas with European roads/highway plus human settlements (patches) at either side of the road are less permeable to bear movement | In Europe, more than a quarter of the lands are situated at an approximate distance of 500m next to the infrastructure, and almost 50% at less that 1.5km^21^. Romania presents a less dense of road infrastructure that Central Europe. Road with high traffic, likewise human settlements can change/block natural routes used of the individuals^22^ and can generate gene flow reduction^23^, together with rivers acting as barriers to movement^6^. | A simple dichotomy of no-roads (value 1) and roads/human settlements (*roadloc*) was applied with five R_max_ values (see aspect). |
| **Human settlements** | The limitative factor *human settlements* have been analysed by considering two categories: ^24^ with human settlements (restricting bears movement) and ^24^ no human settlements (not restricting the bear movement) | Areas inhabited by humans are generally avoided by the brown bears, anthropogenic activities contributing likewise in habitat quality degradation and routes changes^4,18,25^. However, brown bears are using large habitat surfaces were the human footprint is reduced^7,19^ | A value of 1 to no human settlements, while for human settlements we assigned like above series of different maximum resistances (R_max_). |

**Figure S1** Output from Structure Harvester – Mean of estimated Ln probability of data

Bayesian clustering analysis implemented in STRUCTURE using LnPr (*X*|*K*) method indicated *K*=1. The blue dot in the left to which corresponds the value -9000. We infer the brown bears as one population.

**Figure S2** The result from Structure Harvester – Delta K = 2

Estimated number of clusters inferred using Bayesian clustering analysis performed with STRUCTURE HARVESTER for the whole dataset. Blue circles indicate the probability log-likelihood from 20 repeats for each assumed value of *K*. ∆*K* tends to peak at the *K* = 2.


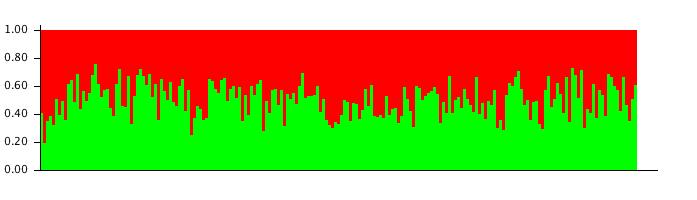


**Figure S3** Stucture results from K=2

Estimated number of clusters inferred using Bayesian clustering analysis. Each bar plot represents an individual. The clusters are not differentiating in the Bayesian histogram generated for ∆*K=2*, thus concluding that a single populations exists

*
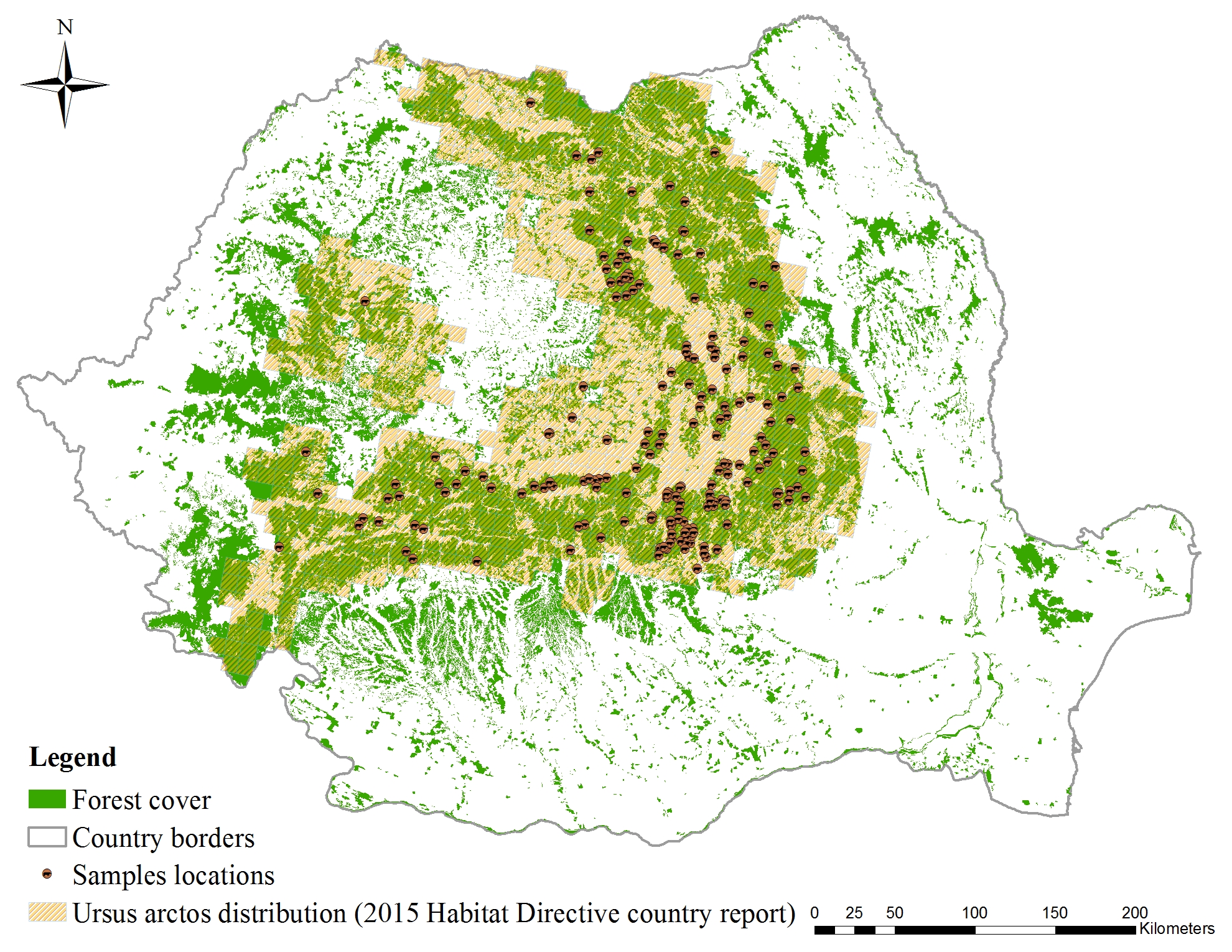
*

**Figure S4** Sampling locations (GPS points) across Romanian Carpathians

Outlined brown cells (10x10 km) represent brown bears distribution (Habitat Directive country report, 2015), green color represents forest cover, while brown points represents samples locations.


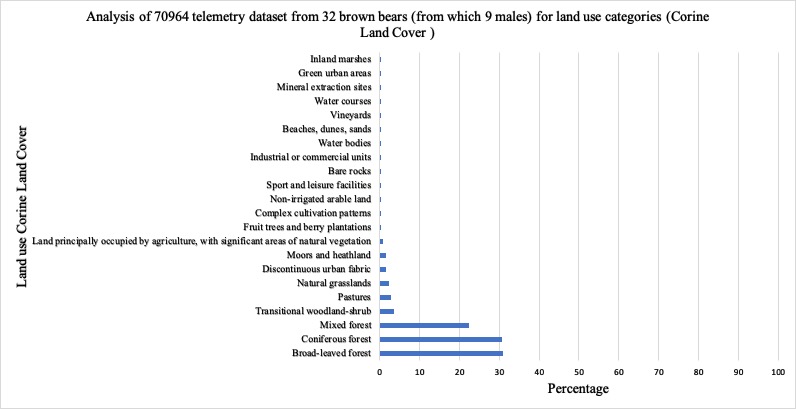


**Figure S5** Analysis of 70964 telemetry dataset from 32 brown bears for land categories, resulting a high preference for forest (broad-leaved forest, coniferous forest and mixed forest)


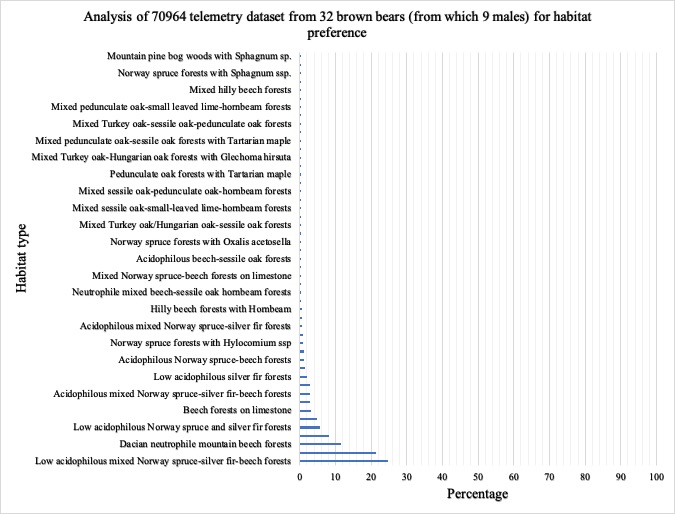


**Figure S6** Analysis of 70964 telemetry dataset from 32 brown bears for habitat types, resulting a general preference for mixed Norway spruce-silver-fir-beech forest and beech forest


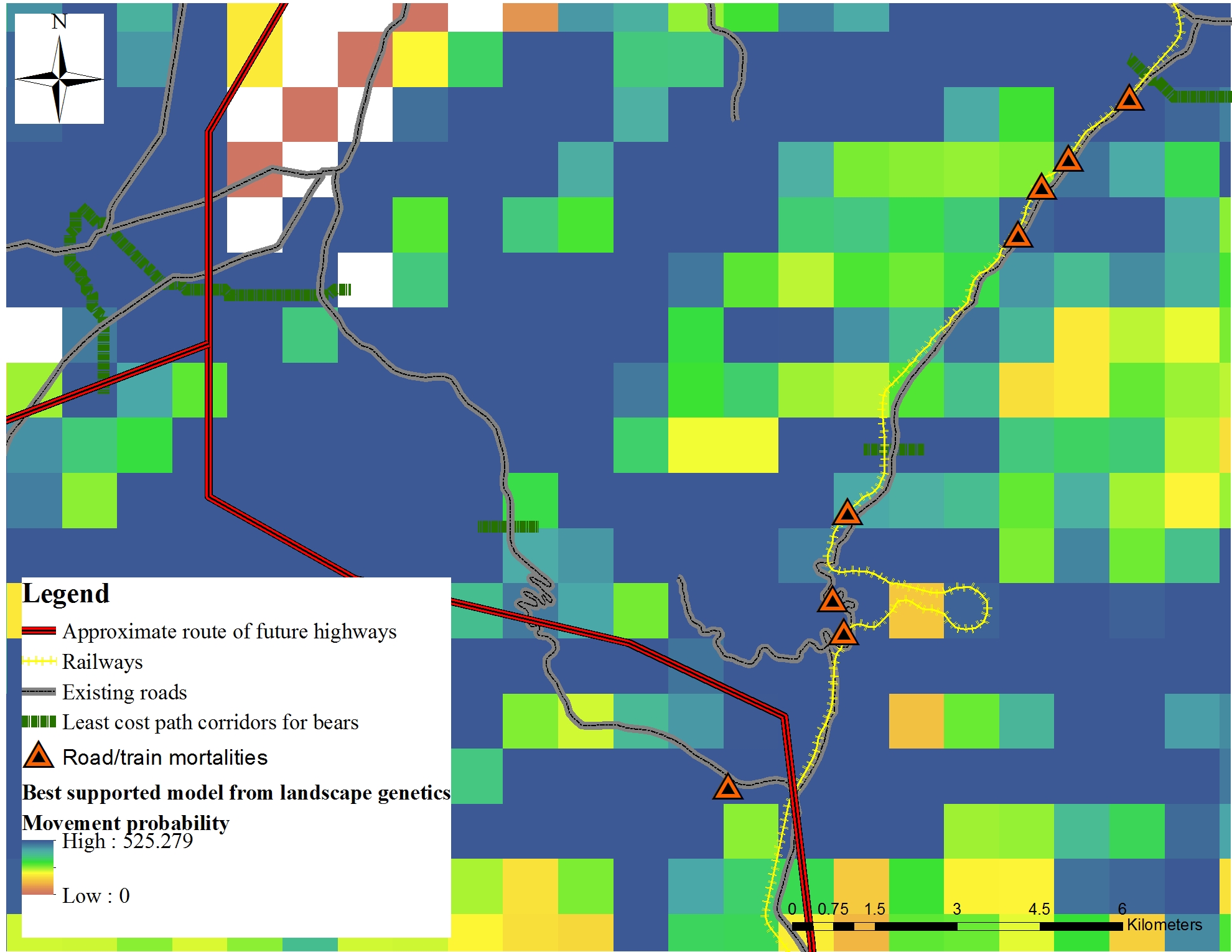


**Figure S7** An example of a wildlife corridor from Prahova Valley (first large wildlife corridor from bottom see Fig. 3b) confirmed by road/train brown bear mortalities and least cost path modelling

References

1 Castillo, J. A., Epps, C. W., Davis, A. R. & Cushman, S. A. Landscape effects on gene flow for a climate-sensitive montane species, the American pika. *Molecular ecology* **23**, 843-856, doi:10.1111/mec.12650 (2014).

2 Wiegand, T., Naves, J., Garbulsky, M. F. & Fernandez, N. Animal habitat quality and ecosystem functioning: exploring seasonal patterns using NDVI. *Ecol. Monogr* **78**, 87–103 (2008).

3 Moe, T. F., Kindberg, J., Jansson, I. & Swenson, J. E. Importance of diel behaviour when studying habitat selection: examples from female Scandinavian brown bears (Ursus arctos). *Canadian Journal of Zoology* **85**, 518-525 (2007).

4 Ziółkowska, E. *et al.* Assessing differences in connectivity based on habitat versus movement models for brown bears in the Carpathians. *Landscape Ecology* **31**, 1863-1882, doi:10.1007/s10980-016-0368-8 (2016).

5 Fernández, N., Selva, N., Yuste, C., Okarma, H. & Jakubiec, Z. Brown bears at the edge: Modeling habitat constrains at the periphery of the Carpathian population. *Biological Conservation* **153**, 134-142, doi:10.1016/j.biocon.2012.04.013 (2012).

6 Koen, E., Bowman, J. & Wilson, P. Isolation of peripheral populations of Canada lynx. *Canadian Journal of Zoology* **93**, doi:10.1139/cjz-2014-0227 (2015).

7 Mateo-Sánchez, M. C. *et al.* Estimating effective landscape distances and movement corridors: comparison of habitat and genetic data. *Ecosphere* **6**, art59, doi:10.1890/es14-00387.1 (2015).

8 McCune, B. & Keon, D. Equations for Potential Annual Direct Incident Radiation and Heat Load. *Journal of Vegetation Science* **13**, 603-606 (2002).

9 Spear, S. F. & Storfer, A. Landscape genetic structure of coastal tailed frogs (Ascaphus truei) in protected vs. managed forests. *Molecular ecology* **17**, 4642-4656, doi:10.1111/j.1365-294X.2008.03952.x (2008).

10 Russo, I.-R. M., Sole, C. L., Barbato, M., von Bramann, U. & Bruford, M. W. Landscape determinants of fine-scale genetic structure of a small rodent in a heterogeneous landscape (Hluhluwe-iMfolozi Park, South Africa). *Scientific Reports* **6**, 29168, doi:10.1038/srep29168 <https://www.nature.com/articles/srep29168#supplementary-information> (2016).

11 Reed-Eckert, M., Meaney, C. & Beauvais, D. G. P. Species assessment for grizzly (brown) bear (Ursus arctos) in Wyoming. *United States Department of the Interior Bureau of Land Management Wyoming State Office* (2004).

12 Munro, R. H. M., Nielsen, S. E., Price, M. H., Stenhouse, G. B. & Boyce, M. S. Seasonal and diel patterns of grizzly bear diet and activity in West-central Alberta. *Journal of Mammalogy* **87**, 1112-1121 (2006).

13 Castillo, J. A., Epps, C. W., Davis, A. R. & Cushman, S. A. Supporting - Landscape effects on gene flow for a climate-sensitive montane species, the American pika.

14 Nagy, K. A. Field metabolic rate and body size. *J Exp Biol* **208**, 1621-1625, doi:10.1242/jeb.01553 (2005).

15 Cushman, S. A., McKelvey, K. S., Hayden, J. & Schwartz, M. K. Gene Flow in Complex Landscapes: Testing Multiple Hypotheses with Causal Modeling. *The American naturalist* **168**, 486-499 (2006).

16 Gaines, W. L., Lyons, A. L., Lehmkuhl, J. F. & Raedeke, K. J. Landscape evaluation of female black bear habitat effectiveness and capability in the North Cascades, Washington. *Biological Conservation* **125**, 411-425, doi:10.1016/j.biocon.2005.03.023 (2005).

17 Nielsen, S. E., Stenhouse, G. B. & Boyce, M. S. A habitat-based framework for grizzly bear conservation in Alberta. *Biological Conservation* **130**, 217-229, doi:10.1016/j.biocon.2005.12.016 (2006).

18 Martin, J. *et al.* Brown bear habitat suitability in the Pyrenees: transferability across sites and linking scales to make the most of scarce data. *Journal of Applied Ecology* **49**, 621-631, doi:10.1111/j.1365-2664.2012.02139.x (2012).

19 Apps, C. D., McLellan, B. N., Woods, J. G., Proctor, M. F. & Gehrt. Estimating Grizzly Bear Distribution and Abundance Relative to Habitat and Human Influence. *Journal of Wildlife Management* **68**, 138-152, doi:10.2193/0022-541x(2004)068[0138:egbdaa]2.0.co;2 (2004).

20 Cushman, S. A., Wasserman, T. N., Landguth, E. L. & Shirk, A. J. Re-Evaluating Causal Modeling with Mantel Tests in Landscape Genetics. *Diversity* **5**, 51-72, doi:10.3390/d5010051 (2013).

21 Torres, A., Jaeger, J. A. & Alonso, J. C. Assessing large-scale wildlife responses to human infrastructure development. *Proceedings of the National Academy of Sciences of the United States of America* **113**, 8472-8477, doi:10.1073/pnas.1522488113 (2016).

22 Waller, J. S. & Servheen, C. Effects of transportation infrastructure on grizzly bears in Northwestern Montana. *The Wildlife Society* **69**, 985-1000, doi:10.2193/0022-541X(2005)069[0985:EOTIOG]2.0.CO;210.2193/0022-541X (2005).

23 Riley, S. P. *et al.* A southern California freeway is a physical and social barrier to gene flow in carnivores. *Molecular ecology* **15**, 1733-1741, doi:10.1111/j.1365-294X.2006.02907.x (2006).

24 Chapron, G. *et al.* Recovery of large carnivores in Europe’s modern human-dominated landscapes. *Science* **346**, doi:10.1594/ (2014).

25 Ordiz, A., Stoen, O. G., Delibes, M. & Swenson, J. E. Predators or prey? Spatio-temporal discrimination of human-derived risk by brown bears. *Oecologia* **166**, 59-67, doi:10.1007/s00442-011-1920-5 (2011).
